# Supplementary material for: SARS-CoV-2 Mpro Protease Variants of Concern Display Altered Viral Substrate and Cell Host Target Galectin-8 Processing but Retain Sensitivity toward Antivirals
Source: ACS Cent Sci. 2023 Mar 21;9(4):696–708. doi: 10.1021/acscentsci.3c00054 (PMC10042146; doi:10.1021/acscentsci.3c00054)
Supplement: Supplementary file 1 — oc3c00054_si_001.pdf [file oc3c00054_si_001.pdf]

Supplementary Materials for

SARS-CoV-2 M^{pro} protease variants of concern display altered viral substrate and cell host target Gal8 processing but retain sensitivity towards antivirals

Sizhu Amelia Chen[1,2], Elena Arutyunova[1,2], Jimmy Lu[1,2], Muhammad Bashir Khan[1], Wioletta Rut[3], Mikolaj Zmudzinski[3], Shima Shahbaz[4], Jegan Iyyathurai[1,2], Eman Moussa[1], Zoe Turner[1], Bing Bai[5,6], Tess Lamer[7], James A. Nieman[5,6], John C. Vederas[7], Olivier Julien[1], Marcin Drag[3], Shokrollah Elahi[4], Howard S. Young[1], M. Joanne Lemieux[1,2*]

Affiliations

1. Department of Biochemistry, University of Alberta, Edmonton, AB, T6G 2H7, Canada, joanne.lemieux@ualberta.ca.
2. Li Ka Shing Institute of Virology, University of Alberta, Edmonton, AB, T6G 2E1, Canada.
3. Department of Chemical Biology and Bioimaging, Wroclaw University of Science and Technology, Wroclaw, 50-370, Poland.
4. Department of Dentistry & Dental Hygiene, University of Alberta, Edmonton, AB, T6G 2E1, Canada.
5. Li Ka Shing Applied Virology Institute, University of Alberta, Edmonton, AB, T6G 2E1, Canada.
6. Department of Medical Microbiology and Immunology, University of Alberta, Edmonton, AB, T6G 2E1, Canada.
7. Department of Chemistry, University of Alberta, Edmonton, AB, T6G 2G2, Canada.

Correspondence to: mlemieux@ualberta.ca

This PDF file includes:

Figures. S1 to S9
Tables S1 to S2

Table of Contents:

1. **Methods** **S2-S7**
2. **References** **S8**
3. **Supplemental figures** **S9-S25**

Methods

Mutation identification and prevalence calculations.

Only "high coverage" sequences were selected for analysis. The prevalence was calculated as a percentage of a certain mutation compared to the amount of all mutations in *nsp5* gene in a particular variant.

Mutagenesis and protein purification.

SARS-CoV-2 M^{pro}. M^{pro} SARS-CoV-2 gene with N-terminal His-SUMO tag in pET SUMO expression vector (Invitrogen) was used for protein production. The mutations were introduced into protein gene using Site-Directed mutagenesis kit (Agilent, Canada). Purification of SARS-CoV-2 M^{pro} wild type and variants were performed as described earlier [1].

Gal-8. Gal-8-6X His gene in pET vector was transformed into Rosetta (DE3) *E.coli* cells and the protein was expressed for 18 hours at 18 ℃. The harvested cells were resuspended in 50 mM HEPES, pH 7.0, 300 mM NaCl, 5 mM Imidazole buffer, lysed by sonication and the protein was purified using.

Gal-9. Gal-9-6X His gene in pBad vector was transformed into TOP-10 *E. coli* cells and the protein was expressed for18 hours at 18 ℃. The harvested cells were resuspended in 50 mM Tris-HCl, pH 7.5, 300 mM NaCl, 5% glycerol, 5mM Imidazole buffer and lysed by sonication followed by d centrifugation step at 17,000 g for 30 min and Ni-NTA affinity chromatography. The final protein sample was concentrated using Amicon Ultra-15 (MWCO 10 kDa) and flash frozen.

HyCoSuL screening

Substrate specificity profile of SARS-CoV-2 M^{pro} wild-type and the mutants was determined in the same manner as described previously [2]. Library screening was performed using a spectrofluorometer (Molecular Devices Spectramax Gemini XPS) on 96-well plates. Enzymes were diluted in assay buffer (20 mM Bis-Tris, 1 mM DTT, pH 7.4) and added to plate wells. Assay conditions were 1 μL of substrate in DMSO and 99 μL of enzyme. Substrate hydrolysis was measured for 60 min at the appropriate wavelength (λ_{ex} = 355 nm, λ_{em} = 460 nm).

FRET-based SARS-CoV-2 M^{pro} activity assay.

FRET-based cleavage assays with synthesized fluorescent substrate of SARS-CoV-2 M^{pro} (Abz-SVTLQ↓SG-Tyr(NO_2)-R) were conducted as described previously [3].

SARS-CoV-2 M^{pro} activity assay with Gal-8.

Gel-based end point activity assay using recombinant Gal-8 was conducted using 14 µM of substrate and 2.5 µM of SARS-CoV-2 M^{pro} WT or variants in activity buffer (20 mM Bis–Tris, pH 7.8, 1 mM DTT). The mixture was incubated at 37 ℃, 30 µL aliquots were taken out at specific time points, the reaction was stopped by adding 5 µM of GC376 inhibitor and then SDS-sample buffer. Protein samples were analysed by SDS-PAGE and digitization was carried out with ImageQuant LAS 4000 (GE Healthcare, USA).

Differential scanning fluorimetry.

DFS was performed using 6 µM of protease with a final concentration of 5X for SyproOrange dye (Thermo Fisher Scientific, USA) in 50 mM Tris-HCl, pH 8.0, 150 mM NaCl. All samples were run in duplicate. The thermal scan was conducted from 25 to 95 ℃, at 0.5 ℃/min (ViiA 7 Real-Time PCR System, ThermoFisher). The melting point (T_m) was calculated by fitting the raw fluorescence data over the temperature using the Boltzmann equation in GraphPad Prism program (Prism 9, GraphPad Software, USA).

Human subject's ethics statement.

This study was approved by the Research Ethics Board at the University of Alberta (protocol # Pro000046064). A written informed consent form was obtained from the study participants.

Sample processing.

Peripheral blood mononuclear cells (PBMCs) were isolated from the blood using Ficoll-Paque gradients. Cell cultures were performed in RPMI 1640 (Sigma-Aldrich) supplemented with 10% FBS (Sigma-Aldrich) and 1% penicillin/streptomycin (Sigma-Aldrich).

Cell culture and Flow cytometry.

Human cell antigens and cytokines fluorophore-labelled antibodies were purchased from BD Biosciences and Thermo Fisher Scientific and used for anti-CD3 (SK7), anti-CD4 (RPA-T4), anti-CD8 (RPA-T8), anti-CD14 (61D3), anti-CD19 (HIB19), anti-TNF-α (MAB11), and anti-IL-6 (MQ2-13A5). Live dead staining was used to exclude dead cells. Surface and intracytoplasmic cytokine staining (ICS) was performed according to our previous reports [4-6]. For ICS, PMBCs were cultured and stimulated with LPS (1 µg/mL), Gal-8 full length or truncated (1 µl/ml) for 6 hr in the presence of Brefeldin A (1 µl/ml). Cells were fixed and permeabilized and acquired on an LSRFortessa-SORP and analyzed using the FlowJo software (version 10).

ELISA

PBMCs were cultured in the absence or presence of Gal-8 full length or truncated (1 µl/ml) for overnight. The concentration of TNF-α in the culture supernatants was measured using an ELISA kit (R&D Systems).

Statistical analysis

The non-parametric tests such as the Mann-Whitney U-test or Kruskal–Wallis one-way analysis of variance was used. The P-values are shown in the graphs and measures are expressed as mean ± SEM and P-value < 0.05 was considered to be statistically significant.

Crystallography

SARS-CoV-2 M^{pro} wild-type and variants were dialyzed against 5 mM Tris-HCl pH 8.0, 10 mM NaCl buffer overnight at 4 °C. Protein was concentrated to 8 mg/ml. SARS-CoV-2 M^{pro}, wild-type and variants, were subjected to the JCSG plus and PACT crystallization screen (Molecular Dimensions) with hits identified under several conditions. The best crystals for SARS-CoV-2 M^{pro} A7T and K90R were observed with sitting drop at room temperature at a ratio of 1:1 with mother liquor 0.2 M lithium chloride, 0.1 M HEPES pH 7.0 and 20% w/v PEG6000. The best crystals for SARS-CoV-2 M^{pro} E47N were observed with sitting drop at room temperature at a ratio of 1:1 with mother liquor 0.2 M sodium fluoride, 20% w/v PEG3350. The best crystals for SARS-CoV-2 M^{pro} E47K was observed using sitting drop at room temperature at a ratio of 1:1 with mother liquor 0.2 sodium thiocyanate, 0.1 M Bis-Tris propane pH 7.5, 20% w/v PEG3350. The best crystals for SARS-CoV-2 M^{pro} L50F was observed using sitting drop at room temperature at a

ratio of 1:1 with mother liquor 0.1 M MMI pH 9.0, 25% w/v PEG1500. The best crystals for SARS-CoV-2 M^{pro} L89F were observed using sitting drop at room temperature at a ratio of 1:1 with mother liquor 0.2 M sodium formate, 0.1 M Bis-Tris Propane pH 6.5, 20% w/v PEG3350. The best crystals for SARS-CoV-2 M^{pro} P132H were observed using sitting drop at room temperature at a ratio of 1:1 with mother liquor 0.2 ammonium phosphate monobasic, 0.1 M Tris pH 8.5, 50% v/v MPD. The best crystals for SARS-CoV-2 M^{pro} T190I, A260V, G283S and S384G were observed using sitting drop at room temperature at a ratio of 1:1 with mother liquor 0.2 M sodium chloride, 0.1 M HEPES pH 7.0, 20% w/v PEG6000. Prior to freezing, crystals were incubated with 22% glycerol as a cryoprotectant. Data collection took place at Stanford Synchrotron Radiation Lightsource (SSRL) (Menlo Park, CA) beamline 12-2 with Blu-Ice using the Web-Ice interface and at Canadian Light Source, Inc. (CLSI) beamline 08B1-1 using MxDC.

Diffraction data collection, phase determination, model building, and refinement

Diffraction data sets for SARS-CoV-2 M^{pro} A7T, L50F and T190I were collected at 100 K in a cold nitrogen stream using CLSI beamline 08B1-1 coupled to PILATUS 6M. Diffraction data sets for SARS-CoV-2 M^{pro} E47N, E47K, L89F, K90R, T190I, A260V, G283S and S284G were collected at 100K in a cold nitrogen stream using SSRL beamline 12-2 at a wavelength of 0.979460, equipped with an Eiger 16 M Pixel Array detector. Multiple data sets were collected per variant with the best selected based on data statistics. X-ray detector Software (XDS) and Scala were used for processing of the data sets. All structures were determined by molecular replacement, with the crystal structure of the free enzyme of the SARS-CoV-2 M^{pro} (PDB entry 6WTM) as a search model, using the Phaser program from Phenix, version v1.19.2-4158. Refinement of all eleven structures were performed with phenix.refine in Phenix software. Statistics of diffraction, data processing and model refinement are given in Supplementary Table 3. The model was inspected with Ramachandran plots and final models displayed using PyMOL molecular graphics software (Version 2.5.2 Schrödinger, LLC.)

In-gel Trypsin Digestion

SARS-CoV-2 M^{pro} and Gal-8 were separated after the cleavage assays by 14% SDS-PAGE. The gels were fixed for 20 minutes (50% ethanol, 2% phosphoric acid), washed twice for 20 minutes each (ddH_2O) and stained overnight with blue-sliver coomassie stain (20% ethanol, 10%

phosphoric acid, 750 mM ammonium sulphate, 0.12% Coomassie Blue G-250) and washed twice for 10 minutes each (ddH_2O). Each lane was separated into 2 fractions and cut into 1 mm pieces for analysis. The gel bands were transferred to a round bottom 96-well plate and to each well 200 µl of destaining solution (50 mM ammonium bicarbonate, 50% acetonitrile) was added. The plate was incubated at 37 °C for 10 minutes, the solution was removed, and the distaining was repeated 3 times and then the solution was replaced with acetonitrile. The plate was incubated again at 37 °C for 10 minutes. The dehydration was repeated until the gel bands became white (2 times) and the sample were dried at 37 °C for 10 minutes. The gel bands were rehydrated with 200 µl of reducing solution (100 mM ammonium bicarbonate, 11.4 mM beta-mercaptoethanol) and incubated at 37 °C for 30 minutes. The reducing solution was removed, 200 µl of alkylating solution (100 mM ammonium bicarbonate, 10 mg/mL iodoacetamide) was added, the gel bands were incubated at 37 °C for 30 minutes and washed with 200 µl of 100 mM ammonium bicarbonate at 37 °C for 10 minutes twice, dehydrated in 200 µl acetonitrile at 37 °C for 10 minutes and dried at 37 °C for 15 minutes. The gel bands were trypsinized (100 µl of 100 mM ammonium bicarbonate and 6 µg/ml Sequence Grade Modified Trypsin, Promega Inc.) overnight. The solutions containing tryptic peptides were transferred to a round bottom 96-well plate. Tryptic peptides were further extracted from the gel bands with extraction solution (2% acetonitrile, 1% formic acid) and incubated at 37 °C for 1 hour. A final extraction was conducted using 50% acetonitrile and 0.5% formic acid and incubated at 37 °C for 1 hour. All solutions containing tryptic peptides were transferred to a round bottom 96-well plate and freeze-dried under vacuum overnight. The samples were resuspended (1 fraction per lane) in 0.1% formic acid before analysis by liquid chromatography tandem mass spectrometry (LC-MS/MS).

Mass spectrometry analysis

Samples were analyzed using a nanoflow-HPLC (Thermo Scientific EASY-nLC 1200 System) coupled to an Orbitrap Fusion Lumos Tribrid Mass Spectrometer (Thermo Fisher Scientific). The peptide mixture underwent reverse phase separation by an analytical column (2 µm, 100 Å, 50 µm × 15 cm, PepMap RSLC C18: Thermo Fisher Scientific). Peptides were eluted over a 45 minute (whole assay) or a 30 minute (individual protein fragment bands) linear gradient from 0 to 36.8% acetonitrile in 0.1% formic acid. Data analysis was conducted using ProteinProspector (v5.22.1) against a concatenated database of the Homo sapien proteome (SwissProt.2017.11.01.

random.concat). The search parameter included digestion by TrypsinPro with non-specificity at the N-termini, a maximum of 3 missed trypsin cleavages, a precursor change range of +2 +3 +4, a precursor mass tolerance of 15 ppm, a fragment mass tolerance of 0.8 Da, carbamidomethylation of Cys (constant modification), and oxidation of Met and deamidation of Asn and Gln (variable modifications). A decoy database search was conducted to evaluate the false-positive rates. The target false discovery rate was set at 2% at the protein level and 1% at the peptide level.

References

1. Arutyunova, E.; Khan, M. B.; Fischer, C.; Lu, J.; Lamer, T.; Vuong, W.; van Belkum, M. J.; McKay, R. T.; Tyrrell, D. L.; Vederas, J. C.; Young, H. S.; Lemieux, M. J., N-Terminal Finger Stabilizes the S1 Pocket for the Reversible Feline Drug GC376 in the SARS-CoV-2 M(pro) Dimer. *J Mol Biol* **2021,** *433* (13), 167003.

2. Rut, W.; Groborz, K.; Zhang, L.; Sun, X.; Zmudzinski, M.; Pawlik, B.; Wang, X.; Jochmans, D.; Neyts, J.; Mlynarski, W.; Hilgenfeld, R.; Drag, M., SARS-CoV-2 M(pro) inhibitors and activity-based probes for patient-sample imaging. *Nat Chem Biol* **2021,** *17* (2), 222-228.

3. Vuong, W.; Khan, M. B.; Fischer, C.; Arutyunova, E.; Lamer, T.; Shields, J.; Saffran, H. A.; McKay, R. T.; van Belkum, M. J.; Joyce, M. A.; Young, H. S.; Tyrrell, D. L.; Vederas, J. C.; Lemieux, M. J., Feline coronavirus drug inhibits the main protease of SARS-CoV-2 and blocks virus replication. *Nat Commun* **2020,** *11* (1), 4282.

4. Elahi, S.; Shahbaz, S.; Houston, S., Selective Upregulation of CTLA-4 on CD8+ T Cells Restricted by HLA-B*35Px Renders them to an Exhausted Phenotype in HIV-1 infection. *PLoS Pathog* **2020,** *16* (8), e1008696.

5. Shahbaz, S.; Dunsmore, G.; Koleva, P.; Xu, L.; Houston, S.; Elahi, S., Galectin-9 and VISTA Expression Define Terminally Exhausted T Cells in HIV-1 Infection. *J Immunol* **2020,** *204* (9), 2474-2491.

6. Shahbaz, S.; Xu, L.; Sligl, W.; Osman, M.; Bozorgmehr, N.; Mashhouri, S.; Redmond, D.; Perez Rosero, E.; Walker, J.; Elahi, S., The Quality of SARS-CoV-2-Specific T Cell Functions Differs in Patients with Mild/Moderate versus Severe Disease, and T Cells Expressing Coinhibitory Receptors Are Highly Activated. *J Immunol* **2021,** *207* (4), 1099-1111.

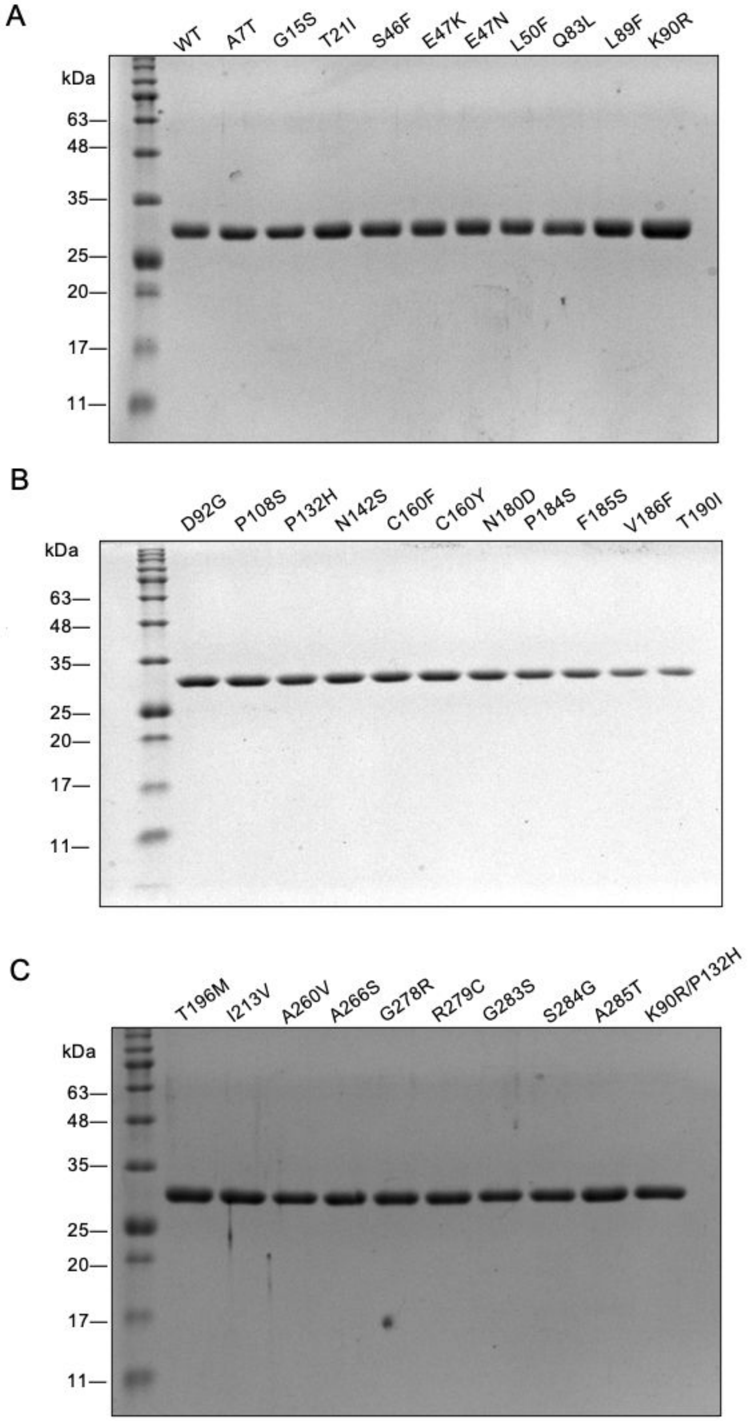

Figure S1. SDS-PAGE of recombinant SARS-CoV-2 M^{pro} wild-type and the mutants. (A-C). The wild type and the VOC mutants of M^{pro} were purified using Ni-NTA resin, followed by SUMO tag removal and Size Exclusion Chromatography on Superdex75 (10/300) column.

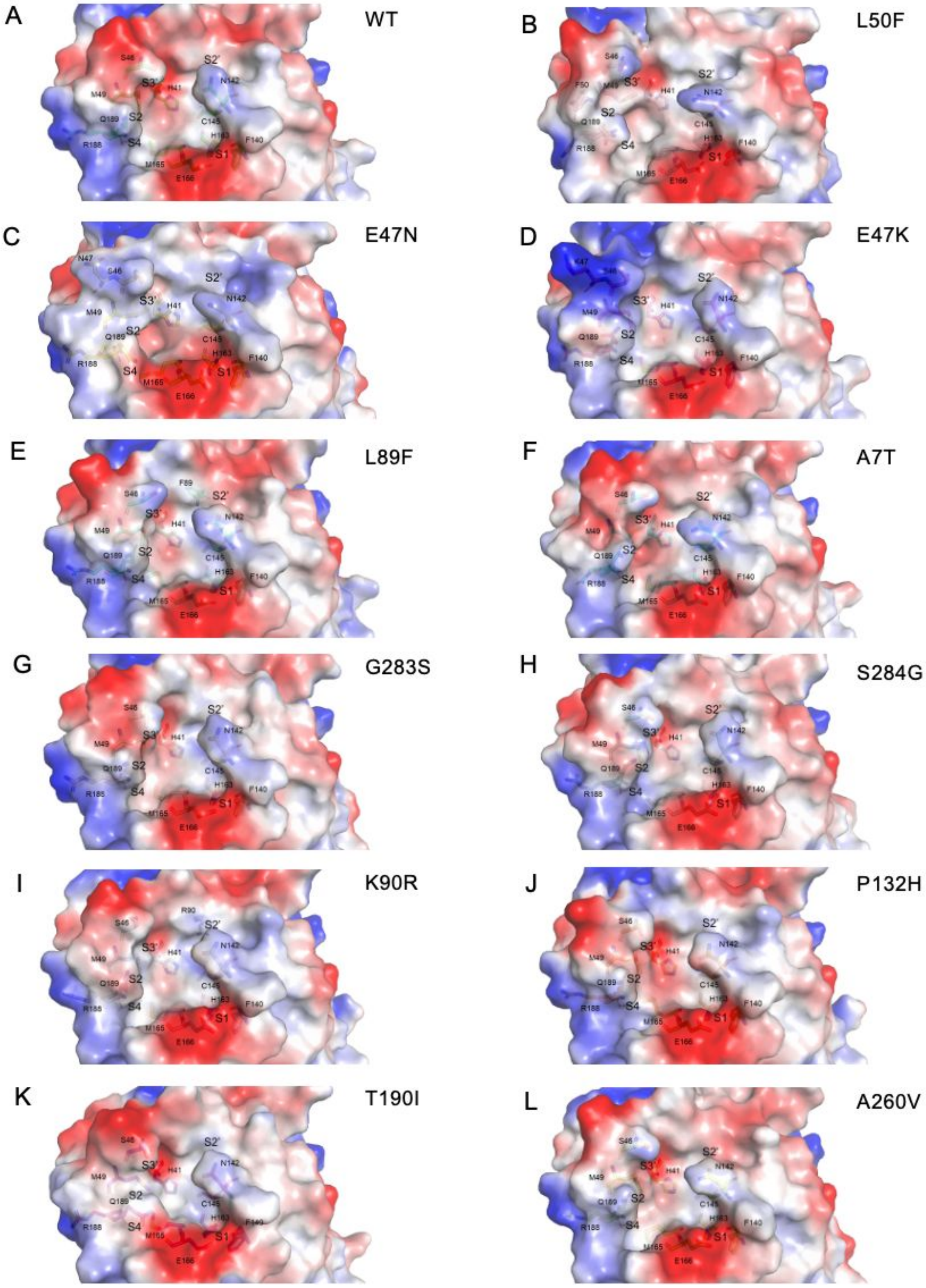

Figure S2. Molecular surface representations showing the electrostatic surface potentials of M^{pro} wild type (A) and the mutants (B-L). All residues in the active site pocket are labelled.

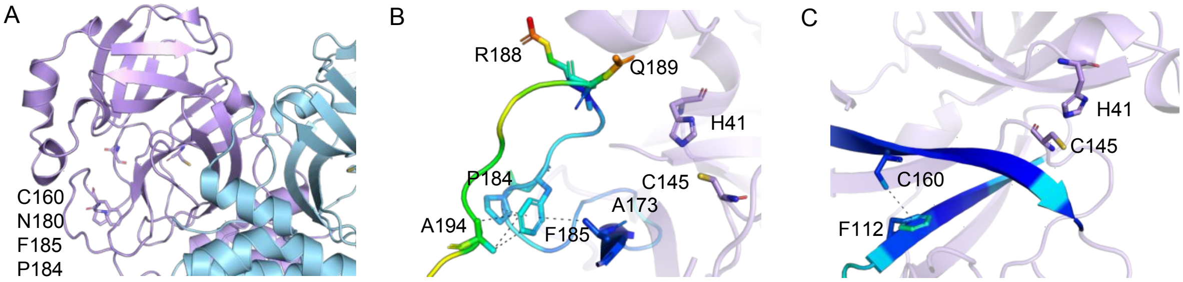

Figure S3. The distribution of mutations with most changes in thermal stability in SARS-CoV-2 M^{pro} molecule (PDB 6WTM). (A) The location of mutations in M^{pro} molecule, which demonstrated the most drastic changes in thermal stability assessed by DSF. (B) and (C) B-factor differences are displayed for the structural elements in a region of M^{pro} molecule where destabilizing mutations were found as a blue–yellow–green color ramp with blue indicating the smallest value (stabilization) and green indicating the largest value (destabilization).

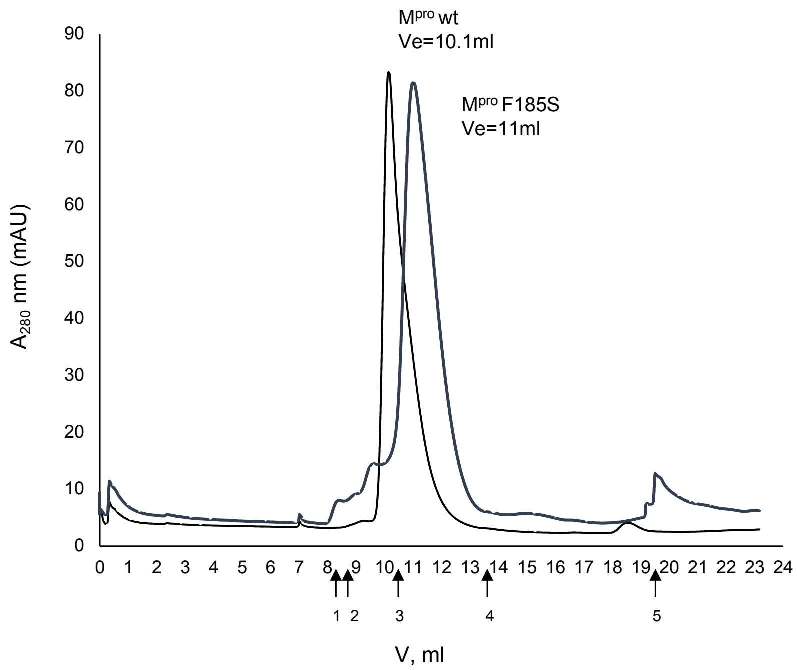

Figure S4. Size exclusion chromatography of M^{pro} wt (black) and M^{pro} F185S (grey) performed on Superdex 75 Increase (10/300) column (Cytiva). Standards: thyroglobin, 8.38 ml, 670 kDa (1.); γ-globulin, 8.78 ml, 158 kDa (2); ovalbumin, 10.67 ml, 44 kDa (3); myoglobin, 13.46 ml, 17 kDa (4); vitamin B12, 19.34 ml, 1.35 kDa.

Ac-Mix-P3-Mix-Gln-ACC

Variants	L-Ala	L-Arg	L-Asn	L-Asp	L-Glu	L-Gln	Gly	L-His	L-Ile	L-Leu	L-Lys	L-Phe	L-Pro	L-Ser	L-Thr	L-Trp	L-Tyr	L-Val
WT	5	22	0	0	0	15	0	7	14	15	33	0	0	0	18	11	8	23
E47K	3	15	0	0	0	0	0	0	15	11	22	0	0	0	21	10	6	26
E47N	0	21	0	0	0	13	0	8	19	14	38	0	0	0	18	12	7	31
L50F	0	0	0	0	0	8	0	0	17	13	22	0	0	0	11	18	0	32
L89F	0	17	0	0	0	0	0	0	17	13	39	0	0	0	17	12	6	27
K90R	0	29	0	0	0	22	0	0	19	23	42	0	0	0	25	19	16	38
P108S	0	0	0	0	0	0	0	0	18	12	31	0	0	0	15	10	3	24
P132H	0	19	0	0	0	0	0	0	16	12	35	0	0	0	17	13	9	24
A260V	0	9	0	0	0	8	0	0	16	11	21	0	0	0	19	10	0	22
G283S	14	13	0	0	0	0	0	0	14	8	29	0	0	0	14	15	0	21
S284G	0	15	0	0	0	7	0	0	10	12	27	0	0	0	17	10	8	21

Ac-P4-Mix-Mix-Gln-ACC

Variant	L-Ala	L-Arg	L-Asn	L-Asp	L-Glu	L-Gln	Gly	L-His	L-Ile	L-Leu	L-Lys	L-Phe	L-Pro	L-Ser	L-Thr	L-Trp	L-Tyr	L-Val
WT	80	0	0	0	0	0	0	0	11	0	0	0	31	29	55	27	0	85
E47K	93	0	0	0	0	0	0	0	0	0	0	0	0	21	35	14	0	82
E47N	73	0	0	0	0	0	0	0	0	0	0	0	13	23	40	15	0	69
L50F	52	0	0	0	0	0	0	0	0	0	0	0	0	0	16	0	0	53
L89F	65	0	0	0	0	0	0	0	0	0	0	0	27	31	42	25	0	69
K90R	77	0	0	0	0	0	0	0	0	0	0	0	0	0	0	0	0	84
P108S	65	0	0	0	0	0	0	0	0	0	0	0	0	0	30	16	0	61
P132H	89	0	0	0	0	0	0	0	0	0	0	0	15	23	38	13	0	92
A260V	55	0	0	0	0	0	0	0	0	0	0	0	0	0	21	0	0	55
G283S	46	0	0	0	0	0	0	0	0	0	0	0	0	22	33	17	0	53
S284G	59	0	0	0	0	0	0	0	0	0	0	0	18	13	35	15	0	58

Relative activity, %

0 20 100

Figure S5. Substrate specificity profile of SARS-CoV-2 M^pro presented as heat maps using substrate library with natural amino acids assessing the P3 and P4 positions with Ac-Mix-P3-Mix-Gln-ACC and Ac-P4-Mix-Mix-Gln-ACC.

Ac-Mix-Mix-P2-Gln-ACC

Variant	D-Ala	D-Arg	D-Asn	D-Asp	D-Gln	D-Glu	D-His	D-Leu	D-Lys	D-Phe	D-Pro	D-Ser	D-Phg	D-Thr	D-Trp	D-Tyr	D-Val	D-hPhe	B-Ala	L-Aze
WT	0	8	0	0	8	0	0	0	0	0	0	0	0	0	6	0	0	7	5	0
E47K	0	6	0	0	0	0	0	0	0	0	0	0	0	0	7	0	0	0	10	0
E47N	0	0	0	0	0	0	4	5	5	0	6	6	0	0	6	0	0	0	9	0
L50F	0	0	0	0	0	0	0	5	0	4	0	0	0	0	8	0	0	6	9	0
L89F	0	0	0	0	0	0	0	4	0	0	0	0	0	0	5	0	0	0	8	0
K90R	0	0	0	0	0	0	6	8	8	7	9	7	6	0	8	6	6	6	13	0
P108S	0	8	0	0	8	0	6	7	0	0	0	0	0	0	8	0	0	0	14	0
P132H	0	0	0	0	0	0	0	0	0	0	0	0	0	0	11	0	0	11	16	0
A260V	7	11	9	4	10	6	8	9	8	6	10	8	7	5	10	7	6	9	16	6
G283S	4	6	5	0	4	11	9	9	4	0	6	8	8	0	9	4	0	3	11	0
S284G	0	8	6	4	7	0	5	6	5	6	9	5	7	4	9	0	6	9	13	5

Variant	L-Hyp	L-Hyp(Bzl)	L-Thz	L-Oic	L-Idc	L-Pip	L-Tic	AC5C	dhAbu	dhLeu	L-Dap	L-Dab	L-Dab(Z)	L-Cit	L-hCit	L-Orn	L-Lys(TFA)	L-Lys(Ac)	L-Lys(2-ClZ)	L-Agp
WT	0	6	5	0	5	6	6	0	2	3	0	0	0	2	6	0	1	0	0	3
E47K	0	8	0	7	11	0	6	0	0	0	0	6	0	0	0	0	0	0	0	6
E47N	0	8	6	6	8	5	7	0	7	5	5	6	5	0	0	0	0	0	0	5
L50F	0	9	0	8	7	0	8	0	5	4	0	2	3	0	4	0	5	0	0	4
L89F	0	7	4	4	0	0	6	0	0	0	0	5	0	0	0	0	0	0	0	0
K90R	6	12	9	11	9	7	9	0	7	7	6	9	7	8	8	8	7	0	0	9
P108S	0	13	5	9	8	7	8	0	0	7	6	9	8	7	7	5	7	0	0	7
P132H	0	15	13	13	14	10	11	0	0	10	0	11	0	0	0	0	0	0	0	9
A260V	8	16	13	11	22	8	10	7	11	8	9	11	10	8	9	8	9	5	0	8
G283S	6	9	6	7	5	4	6	0	6	5	6	6	5	6	5	2	5	4	0	6
S284G	5	12	8	9	11	7	9	4	5	7	5	8	6	6	6	5	7	0	0	6

Variant	L-Agb	L-Arg(Z)$_2$	L-hArg	L-His(3-Bom)	L-Phe(NH$_2$)	L-Phe(guan)	L-Trp(Me)	L-Dht	L-Asp(O-Me)	L-Asp(O-CHx)	L-Asp(O-Bzl)	L-Asp(OAll)	L-Glu(O-Me)	L-Glu(O-CHx)	L-Glu(O-Bzl)	L-Glu(All)	L-Aad	L-Phe(2-F)	L-Phe(3-F)	L-Phe(4-F)
WT	11	0	3	4	5	3	3	9	5	4	6	6	5	5	4	4	0	4	0	4
E47K	15	0	0	0	0	8	10	12	11	11	12	13	11	13	12	11	8	10	10	11
E47N	15	0	5	2	5	4	6	8	6	5	7	7	4	8	6	4	1	4	4	4
L50F	9	0	3	0	7	3	8	17	0	9	0	0	0	12	5	6	0	0	6	7
L89F	11	0	3	0	4	4	7	9	8	0	4	7	7	0	0	6	0	0	0	0
K90R	18	3	9	0	7	5	9	10	5	7	6	6	6	8	7	7	0	4	4	6
P108S	18	0	8	0	7	3	9	10	0	0	0	0	0	8	0	0	0	0	0	0
P132H	21	0	11	0	8	4	6	10	5	6	6	7	5	7	4	3	0	2	6	6
A260V	21	5	11	5	9	9	8	12	9	8	8	9	8	8	11	10	0	0	4	7
G283S	16	4	4	2	7	3	6	13	4	9	6	6	3	6	6	10	0	5	6	6
S284G	17	7	8	4	8	6	8	14	7	8	6	7	8	9	9	5	0	8	8	8

Variant	L-Phe(3,4-F$_2$)	L-Phe(F$_5$)	L-Phe(2-Cl)	L-Phe(3-Cl)	L-Phe(4-Cl)	L-Phe(3,4-Cl$_2$)	L-Phe(4-Br)	L-Phe(3-I)	L-Phe(4-I)	L-Phe(4-Me)	L-3-Pal	L-4-Pal	L-Ala(2-th)	L-Ala(Bth)	L-Abu	L-Abu(Bth)	L-Ser(Ac)	L-Ser(Bzl)	L-hSer	L-Thr(Bzl)
WT	0	2	6	0	3	0	3	3	2	3	3	5	5	0	0	0	6	3	4	3
E47K	8	8	14	0	9	7	7	5	8	7	9	14	11	6	8	6	13	12	7	10
E47N	0	0	4	0	0	0	0	0	0	3	0	6	6	0	0	0	5	8	2	7
L50F	0	0	5	0	0	0	0	0	0	4	0	8	7	0	0	0	0	0	0	0
L89F	0	0	7	0	0	0	0	0	0	0	0	5	5	0	0	0	6	4	0	0
K90R	0	0	7	0	0	0	0	0	1	2	4	7	5	2	0	0	0	5	0	0
P108S	0	0	0	0	0	0	0	0	0	0	0	4	7	0	0	0	0	0	0	0
P132H	0	0	8	0	0	0	0	0	0	0	0	8	6	0	4	0	0	7	0	0
A260V	5	0	8	0	5	0	4	4	6	4	5	9	8	4	0	0	7	6	4	0
G283S	3	0	6	0	0	0	9	0	0	0	0	6	4	0	0	0	0	8	7	6
S284G	0	7	9	4	7	5	6	2	5	6	4	9	9	5	5	0	0	8	4	8

Variant	L-Cys(Bzl)	L-Cys(MeBzl)	L-Cys(4-MeOBzl)	L-Met	L-Met(O)	L-Met(O)$_2$	L-Nle(O-Bzl)	L-Phg	L-hPhe	L-Chg	L-Cha	L-hCha	L-Igl	L-1-Nal	L-2-Nal	L-Bip	L-Bpa	L-2-Aoc	L-hLeu	L-Tle
WT	5	2	3	15	2	4	0	8	7	11	10	0	5	3	3	0	0	2	17	8
E47K	7	6	10	20	8	8	8	14	12	15	13	8	11	10	9	6	8	8	18	17
E47N	6	0	5	14	0	0	0	10	7	11	9	0	6	7	5	0	0	0	15	6
L50F	0	0	0	14	0	0	0	0	0	0	0	0	0	15	0	5	7	0	0	10
L89F	0	0	8	16	0	0	0	6	8	11	10	4	6	5	0	0	4	5	15	8
K90R	0	0	5	15	0	3	4	8	6	11	8	2	7	6	4	4	4	0	13	8
P108S	0	0	6	14	0	0	0	9	0	10	0	0	0	4	0	0	0	0	13	7
P132H	5	0	6	15	0	2	3	8	2	8	5	0	0	7	0	0	0	0	12	4
A260V	7	0	6	15	0	6	6	10	13	11	8	0	0	0	7	0	4	7	12	8
G283S	0	0	4	11	0	0	0	6	4	7	3	0	6	9	0	4	0	0	12	7
S284G	6	4	9	16	0	6	0	9	5	11	6	9	7	6	4	5	6	0	13	9

Figure S6. Substrate specificity profile of SARS-CoV-2 M^{pro} presented as heat maps using substrate library with unnatural amino acids assessing the P2 position with Ac-Mix-Mix-P2-Gln-ACC.

Variant	L-Arg(NO2)	L-Tyr(Me)	L-Tyr(2,6-Cl2-Bzl)	L-Tyr(Bzl)	L-Tyr(2-Br-Z)	L-hTyr	L-hTyr(Me)	L-Nva	L-His(Bzl)	L-2-Abz	L-3-Abz	L-4-Abz	Inp	L-Gla	L-Phe(4-NO2)	Aib	L-hSer(Bzl)	TmbGly	L-Nle
WT	3	4	0	0	6	4	4	12	8	7	10	5	5	5	11	4	5	9	19
E47K	9	10	11	7	14	11	10	19	15	16	23	15	11	13	20	11	12	17	19
E47N	0	5	4	0	8	5	5	12	7	8	14	8	5	5	13	6	0	8	20
L50F	0	4	0	6	12	8	0	13	12	0	8	0	0	0	0	0	8	7	17
L89F	5	0	0	0	8	5	5	13	10	11	13	4	0	0	12	0	0	0	20
K90R	4	6	6	2	10	6	5	13	15	11	17	9	2	4	12	6	7	7	23
P108S	0	6	4	0	9	6	0	11	12	8	12	11	4	0	14	0	7	7	23
P132H	2	6	4	0	9	5	5	12	9	8	13	6	4	0	12	5	7	11	25
A260V	7	3	7	6	8	5	6	13	11	14	15	12	9	9	18	8	6	12	22
G283S	0	5	0	0	9	6	0	9	9	7	12	10	5	0	13	0	7	5	20
S284G	3	5	6	0	9	7	0	14	11	11	14	12	7	0	16	6	8	10	24

Ac-Mix-P3-Mix-Gln-ACC

Variants	D-Ala	D-Arg	D-Asn	D-Asp	D-Gln	D-Glu	D-His	D-Leu	D-Lys	D-Phe	D-Pro	D-Ser	D-Phg	D-Thr	D-Trp	D-Tyr	D-Val	D-hPhe	B-Ala	L-Aze
WT	0	0	0	0	0	0	0	17	0	47	0	0	31	0	29	71	9	8	0	0
E47K	0	0	0	0	0	0	0	18	0	39	0	0	34	0	28	62	8	8	0	0
E47N	0	0	0	0	0	0	0	18	0	43	0	0	36	0	32	74	8	9	0	0
L50F	0	0	0	0	0	0	0	9	0	22	0	0	19	0	15	30	0	7	0	0
L89F	0	0	0	0	0	0	0	12	0	41	0	0	40	0	33	68	0	0	0	0
K90R	0	0	0	0	0	0	0	26	0	48	0	0	42	0	32	78	16	19	0	0
P108S	0	0	0	0	0	0	0	16	0	38	0	0	33	0	26	63	0	0	0	0
P132H	0	0	0	0	0	0	0	20	0	41	0	0	33	0	26	75	0	4	0	0
A260V	0	0	0	0	0	0	0	10	0	33	0	0	24	0	17	55	0	0	0	0
G283S	0	0	0	0	0	0	0	11	0	37	0	0	29	0	24	65	0	0	0	0
S284G	0	0	0	0	0	0	0	14	0	38	0	0	32	0	21	64	0	15	0	0

Variant	L-Hyp	L-Hyp(Bzl)	L-Thz	L-Oic	L-Idc	L-Pip	L-Tic	AC5C	dhAbu	dhLeu	L-Dap	L-Dab	L-Dab(Z)	L-Cit	L-hCit	L-Orn	L-Lys(TFA)	L-Lys(Ac)	L-Lys(2-ClZ)	L-Agp
WT	0	0	0	0	12	0	0	0	0	10	17	43	11	15	9	43	15	16	14	32
E47K	0	0	0	0	0	0	0	0	0	9	14	41	16	14	14	41	19	14	0	33
E47N	0	0	0	0	11	0	0	0	0	10	17	51	21	18	12	48	17	14	0	37
L50F	0	0	0	0	0	0	0	0	0	0	11	47	20	16	20	34	15	15	0	31
L89F	0	0	0	0	5	0	0	0	0	7	17	57	14	12	0	53	13	12	20	43
K90R	0	0	0	0	0	0	0	0	0	17	22	56	18	25	22	58	25	24	12	46
P108S	0	0	0	0	0	0	0	0	0	9	11	47	0	15	0	41	16	13	0	36
P132H	0	0	0	0	0	0	0	0	0	12	15	46	0	14	10	45	16	14	23	38
A260V	0	0	0	0	6	0	0	0	0	0	8	32	8	12	0	34	12	11	26	28
G283S	0	0	0	0	0	0	0	0	0	8	11	43	17	7	0	44	13	14	0	34
S284G	0	0	0	0	0	0	0	0	0	9	16	44	9	13	13	46	14	7	11	31

Variant	L-Agb	L-Arg(Z)2	L-hArg	L-His(3-Bom)	L-Phe(NH2)	L-Phe(guan)	L-Trp(Me)	L-Dht	L-Asp(O-Me)	L-Asp(O-CHx)	L-Asp(O-Bzl)	L-Asp(OAll)	L-Glu(O-Me)	L-Glu(O-CHx)	L-Glu(O-Bzl)	L-Glu(All)	L-Aad	L-Phe(2-F)	L-Phe(3-F)	L-Phe(4-F)
WT	14	0	36	4	9	26	8	38	0	0	0	0	9	9	10	6	0	0	0	0
E47K	15	0	36	0	13	18	0	30	0	0	0	0	0	0	0	0	0	0	0	0
E47N	16	0	41	8	11	27	5	36	0	0	0	0	12	0	0	0	0	0	0	0
L50F	12	0	48	10	15	26	10	40	0	7	0	0	19	8	9	14	0	0	0	0
L89F	13	0	36	0	9	31	3	39	0	0	0	0	11	0	0	0	0	0	0	0
K90R	27	0	53	0	19	25	0	35	0	0	0	0	0	0	0	0	0	0	0	0
P108S	18	0	36	0	14	25	0	32	0	0	0	0	0	0	0	0	0	0	0	0
P132H	14	0	38	0	16	27	9	37	0	0	0	0	0	0	0	0	0	0	0	0
A260V	10	0	32	10	9	21	0	29	0	0	0	0	11	0	0	0	0	0	0	0
G283S	12	0	29	0	9	22	0	35	0	0	0	0	0	8	0	0	0	0	0	0
S284G	16	0	40	0	10	25	0	36	0	0	0	0	14	0	0	11	0	0	0	0

Figure S6 continued. Substrate specificity profile of SARS-CoV-2 M^{pro} presented as heat maps using substrate library with unnatural amino acids assessing the P2 and P3 positions with Ac-Mix-Mix-P2-Gln-ACC and Ac-Mix-P3-Mix-Gln-ACC.

Variant	L-Phe(3,4-F$_2$)	L-Phe(F$_5$)	L-Phe(2-Cl)	L-Phe(3-Cl)	L-Phe(4-Cl)	L-Phe(3,4-Cl$_2$)	L-Phe(4-Br)	L-Phe(3-I)	L-Phe(4-I)	L-Phe(4-Me)	L-3-Pal	L-4-Pal	L-Ala(2-th)	L-Ala(Bth)	L-Abu	L-Abu(Bth)	L-Ser(Ac)	L-Ser(Bzl)	L-hSer	L-Thr(Bzl)
WT	0	0	0	0	0	0	0	0	0	0	8	9	0	0	10	0	0	0	9	7
E47K	0	0	0	0	0	0	0	0	0	0	0	0	0	0	0	0	0	0	0	0
E47N	0	12	0	0	0	0	0	0	0	0	12	12	0	0	13	0	0	0	12	0
L50F	0	8	0	0	0	0	0	0	0	0	14	16	0	0	16	1	0	0	13	6
L89F	0	4	0	0	0	0	0	0	0	0	10	12	0	0	10	0	0	0	11	0
K90R	0	0	0	0	0	0	0	0	0	0	0	0	0	0	0	0	0	0	0	0
P108S	0	0	0	0	0	0	0	0	0	0	0	0	0	0	0	0	0	0	0	0
P132H	0	0	0	0	0	0	0	0	0	0	0	0	0	0	0	0	0	0	0	0
A260V	0	0	0	0	0	0	0	0	0	0	0	0	0	0	0	0	0	0	12	0
G283S	0	0	0	0	0	0	0	0	0	0	10	12	0	0	0	0	0	0	0	0
S284G	0	0	0	0	0	0	0	0	0	0	11	17	0	0	11	0	0	0	10	0

Variant	L-Cys(Bzl)	L-Cys(MeBzl)	L-Cys(4-MeOBzl)	L-Met	L-Met(O)	L-Met(O)$_2$	L-Nle(O-Bzl)	L-Phg	L-hPhe	L-Chg	L-Cha	L-Igl	L-1-Nal	L-2-Nal	L-Bip	L-Bpa	L-2-Aoc	L-Tle	L-Arg(NO$_2$)	L-Tyr(Me)
WT	0	5	11	15	18	28	0	23	3	8	0	0	7	0	0	5	0	100	23	0
E47K	0	0	13	12	8	27	0	26	0	0	0	0	12	0	0	0	0	100	19	0
E47N	0	7	17	20	24	36	0	41	7	15	0	0	11	0	0	7	0	100	28	12
L50F	0	4	25	25	25	39	11	29	18	14	11	0	17	0	0	5	7	100	28	10
L89F	0	0	16	21	22	36	0	28	10	10	3	0	0	0	0	0	0	100	30	0
K90R	0	0	26	15	25	31	0	29	0	0	0	0	0	0	0	0	0	100	23	0
P108S	0	0	0	20	17	36	0	19	0	0	0	0	0	0	0	0	0	100	24	0
P132H	0	0	19	21	21	36	0	20	0	0	0	0	0	0	0	0	0	100	27	0
A260V	0	0	12	13	18	26	0	15	0	12	0	0	17	0	0	0	0	100	22	0
G283S	0	0	15	14	19	35	0	18	11	0	0	0	0	0	0	0	0	100	21	0
S284G	0	0	23	18	25	38	0	23	0	16	0	0	9	8	0	0	0	100	25	6

Variant	L-Tyr(2,6-Cl$_2$-Bzl)	L-Tyr(Bzl)	L-Tyr(2-Br-Z)	L-hTyr	L-hTyr(Me)	L-Nva	L-His(Bzl)	L-2-Abz	L-4-Abz	Inp	L-Gla	L-Phe(4-NO2)	Aib	L-hSer(Bzl)	L-Nle
WT	0	5	11	12	0	9	4	0	0	0	0	0	0	0	9
E47K	0	0	0	14	0	4	0	0	0	0	0	0	0	0	0
E47N	0	0	18	24	9	17	5	0	0	0	0	0	0	0	6
L50F	0	9	18	21	11	17	8	0	0	0	0	0	0	8	0
L89F	0	0	15	19	10	14	0	0	0	0	0	0	0	0	5
K90R	0	0	0	22	22	0	0	0	0	0	0	0	0	0	17
P108S	0	0	13	20	0	0	0	0	0	0	0	0	0	0	0
P132H	0	0	15	13	0	0	0	0	0	0	0	0	0	0	0
A260V	0	6	8	6	9	9	0	0	0	0	0	0	0	0	0
G283S	0	0	12	14	0	17	5	0	0	0	0	0	0	0	0
S284G	0	8	13	17	0	16	0	0	0	0	0	0	0	0	8

Figure S6 continued. Substrate specificity profile of SARS-CoV-2 M^{pro} presented as heat maps using substrate library with unnatural amino acids assessing the P3 position with Ac-Mix-P3-Mix-Gln-ACC.

Ac-P4-Mix-Mix-Gln-ACC

Variant	D-Ala	D-Arg	D-Asn	D-Asp	D-Gln	D-Glu	D-His	D-Leu	D-Lys	D-Phe	D-Pro	D-Ser	D-Phg	D-Thr	D-Trp	D-Tyr	D-Val	D-hPhe	B-Ala	L-Aze
WT	0	0	0	0	0	0	0	0	0	0	0	0	19	0	0	0	0	0	18	32
E47K	0	0	0	0	0	0	0	0	0	0	0	0	13	0	0	0	0	0	0	17
E47N	0	0	0	0	0	0	0	0	0	0	0	0	0	0	0	0	0	0	0	0
L50F	0	0	0	0	0	0	0	0	0	0	0	0	9	0	0	0	0	0	0	0
L89F	0	0	0	0	0	0	0	0	0	0	0	0	0	0	0	0	0	0	0	27
K90R	0	0	0	0	0	0	0	0	0	0	0	0	0	0	0	0	0	0	0	0
P108S	0	0	0	0	0	0	0	0	0	0	0	0	0	0	0	0	0	0	0	0
P132H	0	0	0	0	0	0	0	0	0	0	0	0	0	0	0	0	0	0	0	21
A260V	0	0	0	0	0	0	0	0	0	0	0	0	0	0	0	0	0	0	0	0
G283S	0	0	0	0	0	0	0	0	0	0	0	0	17	0	0	0	0	0	0	26
S284G	0	0	0	0	0	0	0	0	0	0	0	0	9	0	0	0	0	0	0	27

Variant	L-Hyp	L-Hyp(Bzl)	L-Thz	L-Oic	L-Idc	L-Pip	L-Tic	AC5C	dhAbu	dhLeu	L-Dap	L-Dab	L-Dab(Z)	L-Cit	L-hCit	L-Orn	L-Lys(TFA)	L-Lys(Ac)	L-Lys(2-ClZ)	L-Agp
WT	9	19	99	0	0	8	7	0	38	8	0	0	0	0	0	0	0	0	0	0
E47K	0	20	63	0	0	0	0	0	26	0	0	0	0	0	0	0	0	0	0	0
E47N	0	0	66	0	0	0	0	0	24	0	0	0	0	0	0	0	0	0	0	0
L50F	0	0	65	0	0	0	0	0	0	0	0	0	0	0	0	0	0	0	0	0
L89F	0	22	73	0	0	0	0	0	30	0	0	0	0	0	0	0	0	0	0	0
K90R	0	0	66	0	0	0	0	0	0	0	0	0	0	0	0	0	0	0	0	0
P108S	0	17	64	0	0	0	0	0	19	0	0	0	0	0	0	0	0	0	0	0
P132H	0	19	63	0	0	0	0	0	25	0	0	0	0	0	0	0	0	0	0	0
A260V	0	0	61	0	0	0	0	0	15	0	0	0	0	0	0	0	0	0	0	0
G283S	0	8	63	0	11	0	0	0	24	10	0	0	0	0	0	0	0	0	0	24
S284G	0	10	69	0	0	0	0	0	15	0	0	0	0	0	0	0	0	0	0	0

Variant	L-Agb	L-Arg(Z)2	L-hArg	L-His(3-Bom)	L-Phe(NH2)	L-Phe(guan)	L-Trp(Me)	L-Dht	L-Asp(O-Me)	L-Asp(O-CHx)	L-Asp(O-Bzl)	L-Asp(OAll)	L-Glu(O-Me)	L-Glu(O-CHx)	L-Glu(O-Bzl)	L-Glu(All)	L-Aad	L-Phe(2-F)	L-Phe(3-F)	L-Phe(4-F)
WT	25	0	0	0	0	0	0	0	0	0	0	0	0	0	0	10	0	0	0	0
E47K	15	0	0	0	0	0	0	0	0	0	0	0	0	0	0	0	0	0	0	0
E47N	0	0	0	0	0	0	0	17	0	0	0	0	0	0	21	15	0	0	0	0
L50F	0	0	0	0	0	0	0	0	0	0	0	0	0	0	0	0	0	0	0	0
L89F	22	0	0	0	0	0	0	0	0	0	0	0	0	0	0	0	0	0	0	0
K90R	0	0	0	0	0	0	0	0	0	0	0	0	0	0	0	0	0	0	0	0
P108S	22	0	0	0	0	0	0	0	0	0	0	0	0	0	0	0	0	0	0	0
P132H	0	0	0	0	0	0	0	0	0	0	0	0	0	0	0	0	0	0	0	0
A260V	0	0	0	0	0	0	0	8	0	0	0	0	0	0	0	0	0	0	0	0
G283S	0	0	0	0	0	0	0	24	0	0	0	0	0	0	0	0	0	0	0	0
S284G	15	0	0	0	0	0	0	20	0	0	0	0	0	0	0	0	0	0	0	0

Figure S6 continued. Substrate specificity profile of SARS-CoV-2 M^{pro} presented as heat maps using substrate library with unnatural amino acids assessing the P4 position with Ac-P4-Mix-Mix-Gln-ACC.

Variant	L-Phe(3,4-F_2)	L-Phe(F_5)	L-Phe(2-Cl)	L-Phe(3-Cl)	L-Phe(4-Cl)	L-Phe(3,4-Cl_2)	L-Phe(4-Br)	L-Phe(3-I)	L-Phe(4-I)	L-Phe(4-Me)	L-3-Pal	L-4-Pal	L-Ala(2-th)	L-Ala(Bth)	L-Abu	L-Abu(Bth)	L-Ser(Ac)	L-Ser(Bzl)	L-hSer	L-Thr(Bzl)
WT	0	0	0	0	0	0	0	0	0	0	0	0	5	0	100	0	45	8	0	36
E47K	0	0	0	0	0	0	0	0	0	0	0	0	0	0	100	0	38	0	0	36
E47N	0	0	0	0	0	0	0	0	0	0	0	0	0	0	100	0	50	16	17	45
L50F	0	0	0	0	0	0	0	0	0	0	0	0	0	0	100	0	0	0	0	0
L89F	0	0	0	0	0	0	0	0	0	0	0	0	0	0	100	0	41	0	0	45
K90R	0	0	0	0	0	0	0	0	0	0	0	0	0	0	100	0	34	0	0	30
P108S	0	0	0	0	0	0	0	0	0	0	0	0	0	0	100	0	33	0	0	56
P132H	0	0	0	0	0	0	0	0	0	0	0	0	0	0	100	0	49	0	0	30
A260V	0	0	0	0	0	0	0	0	0	0	0	0	0	0	100	0	28	0	0	17
G283S	0	0	0	0	0	0	0	0	0	0	0	0	0	0	100	0	35	0	0	29
S284G	0	0	0	0	0	0	0	0	0	0	0	0	0	0	100	0	33	0	0	25

Variants	L-Cys(Bzl)	L-Cys(MeBzl)	L-Cys(4-MeOBzl)	L-Met	L-Met(O)	L-Met(O_2)	L-Nle(O-Bzl)	L-Phg	L-hPhe	L-Chg	L-Cha	L-hCha	L-Igl	L-1-Nal	L-2-Nal	L-Bip	L-Bpa	L-2-Aoc	L-hLeu	L-Tle
WT	0	0	15	0	0	0	0	0	0	0	0	0	0	0	0	0	0	0	0	50
E47K	0	0	14	0	0	0	0	0	0	0	0	0	0	0	0	0	0	0	0	35
E47N	0	0	27	14	0	0	0	0	0	0	0	0	0	10	0	0	0	0	0	49
L50F	0	0	0	0	0	0	0	0	0	0	0	0	0	0	0	0	0	0	0	0
L89F	0	0	10	0	0	0	0	0	0	0	0	0	0	0	0	0	0	0	0	36
K90R	0	0	0	0	0	0	0	0	0	0	0	0	0	0	0	0	0	0	0	36
P108S	0	0	16	0	0	0	0	0	0	0	0	0	0	0	0	0	0	0	0	38
P132H	0	0	16	0	0	0	0	0	0	0	0	0	0	0	0	0	0	0	0	44
A260V	0	0	0	0	0	0	0	0	0	0	0	0	0	0	0	0	0	0	0	39
G283S	0	0	18	0	0	0	0	0	0	0	0	0	0	0	0	0	0	0	0	28
S284G	0	0	15	0	0	0	0	0	0	0	0	0	0	0	0	0	0	0	0	26

Variant	L-Arg(NO_2)	L-Tyr(Me)	L-Tyr(2,6-Cl_2-Bzl)	L-Tyr(Bzl)	L-Tyr(2-Br-Z)	L-hTyr	L-hTyr(Me)	L-Nva	L-His(Bzl)	L-2-Abz	L-3-Abz	L-4-Abz	Inp	L-Gla	L-Phe(4-NO2)	Aib	L-hSer(Bzl)	TmbGly	L-Nle
WT	0	0	0	0	0	0	0	22	0	69	81	25	0	0	0	0	15	0	0
E47K	0	0	0	0	0	0	0	25	0	50	69	0	0	0	0	0	0	15	0
E47N	0	0	0	0	0	0	0	35	0	63	74	33	0	0	0	0	22	31	0
L50F	0	0	0	0	0	0	0	0	0	0	65	0	0	0	0	0	0	0	0
L89F	0	0	0	0	0	0	0	17	0	58	74	17	0	0	0	0	0	0	0
K90R	0	0	0	0	0	0	0	13	0	46	72	0	0	0	0	0	0	0	0
P108S	0	0	0	0	0	0	0	0	0	55	72	26	0	0	0	0	0	0	0
P132H	0	0	0	0	0	0	0	17	0	47	64	17	0	0	0	0	0	28	0
A260V	0	0	0	0	0	0	0	0	0	52	78	0	0	0	0	0	0	0	0
G283S	0	0	0	0	0	0	0	0	0	48	80	0	0	0	0	0	0	19	0
S284G	0	0	0	0	0	0	0	8	0	41	68	0	0	0	0	0	0	0	0

Figure S6 continued. Substrate specificity profile of SARS-CoV-2 M^{pro} presented as heat maps using substrate library with unnatural amino acids assessing the P4 position with Ac-P4-Mix-Mix-Gln-ACC.

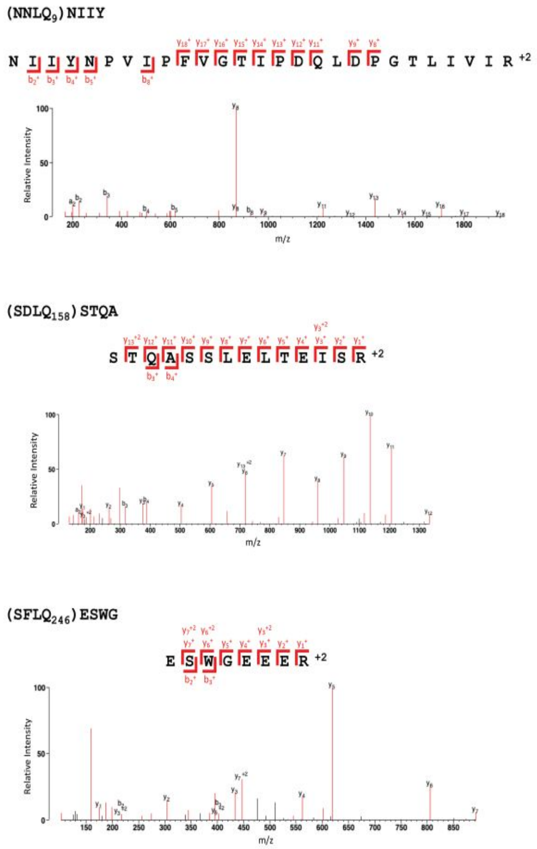

Figure S7. SARS-CoV-2 M^pro cleaves Gal-8 at three sites as observed by mass spectrometry. Following incubation of Gal8 with SARS-CoV-2 Mpro for 2 h, the whole assay was analyzed by mass spectrometry. Three cleavage sites were identified for Gal-8 at LQ_9, LQ_{158}, and LQ_{246}.

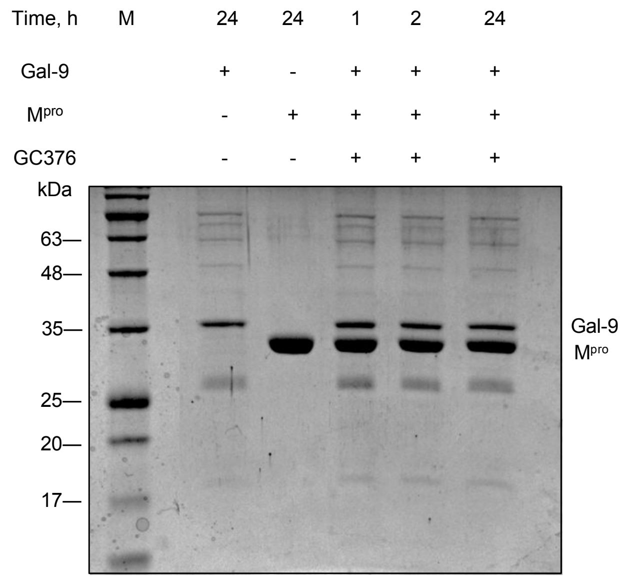

Figure S8. SDS-PAGE-based time-course of Gal-9 cleavage by SARS-CoV-2 M^{pro}. Gal-9 was incubated with the protease at 37°C and reaction was stopped at specific time points with M^{pro} inhibitor, GC376.

A

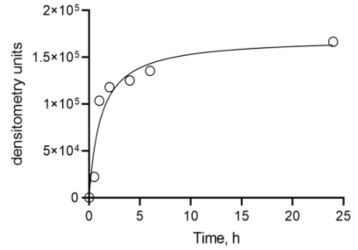

B

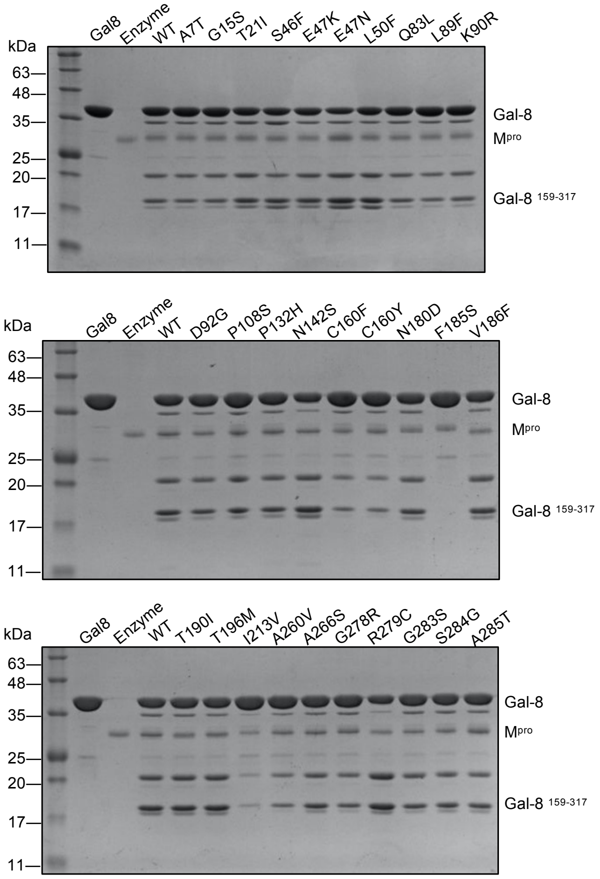

C

M^{pro} mutant	*Gal8 $^{159-317}$ amount, %*
wild type	100
A7T	93.3
G15S	94.0
T21I	140.0
S46F	158.3
E47K	180.8
E47N	139.2
L50F	186.2
Q83L	105.3
L89F	81.9
K90R	104.0
D92G	81.8
P108S	84.2
P132H	89.2
N142S	120.2
C160F	48.6
C160Y	40.9
N180D	108.3
F185S	0
V186F	136.4
T190I	118.0
T196M	113.9
I213V	11.5
A260V	32.2
A266S	71.9
G278R	46.0
R279C	183.0
G283S	83.1
S284G	78.1
A285T	49.9

Figure S9. The cleavage of host cell substrate Gal-8 by SARS-CoV-2 M^{pro} wild type and the mutants. (A) Densitometry analysis of dependence of Gal-8 $^{159-317}$ cleavage product formed on time upon SARS-CoV-2 M^{pro} proteolysis. (B) Gal-8 was incubated with the proteases at 37℃ for 2 hours, the reaction was stopped with M^{pro} inhibitor, GC376 and SDS-PAGE-based densitometry analysis of Gal-8 $^{159-317}$ cleavage product was performed. (C) The results are depicted as a percent of generated Gal-8 $^{159-317}$ product in comparison to Gal-8 cleavage by the wild type.

Table S1. The prevalence of mutations throughout the VOCs calculated as a percentage a certain mutation of all mutations in *nsp5* in a particular variant. Only 'high coverage" sequences were considered at GISAID database (https://gisaid.org).

	Frequency of occurrence,%				
Mutation	*Alpha B.1.1.7*	*Beta B.1.351*	*Gamma P.1*	*Delta B.1.617.2*	*Omicron B.1.1.529*
A7T	0.100	0.004	0.062	0.321	0.010
G15S	0.047	0.035	0.025	0.322	0.011
T21I	1.493	0.164	9.647	0.966	0.082
S46F	0.235	0.004	0.111	0.750	0.006
E47N	8.713	0.000	0.000	0.000	0.000
E47K	0.065	0.008	0.050	0.015	0.003
L50F	0.381	0.093	0.421	0.833	0.011
Q83L	0.005	0.000	0.000	0.016	0.003
L89F	0.720	0.008	2.093	0.307	0.034
K90R	23.672	99.887	16.223	21.764	0.581
D92G	0.320	0.000	0.025	0.184	0.003
P108S	5.379	0.277	2.885	1.873	0.056
P132H	0.011	0.000	0.000	0.131	99.947
N142S	0.000	0.000	0.000	0.007	0.000
C160F	4.109	0.035	0.693	0.086	0.003
C160Y	0.165	0.004	0.136	0.126	0.001
N180D	0.023	0.000	0.000	0.049	0.004
P184S	0.652	0.008	0.186	0.544	0.026
F185S	0.000	0.000	0.000	0.002	0.000
V186F	0.167	0.012	7.207	0.173	0.002
T190I	0.269	0.008	0.111	0.284	0.004
T196M	0.151	0.000	0.025	0.879	0.010
I213V	0.070	0.000	0.037	2.381	0.004
A260V	3.784	0.051	0.793	5.038	0.020
A266S	0.005	0.000	0.000	0.130	0.000
G278R	0.023	0.000	0.000	0.041	0.001
R279C	1.207	0.012	0.644	0.307	0.016
G283S	0.200	0.000	0.062	0.064	0.006
S284G	0.090	0.004	0.037	0.242	0.070
A285T	0.042	0.000	0.916	0.020	0.021
K90R P132H	0.000	0.000	0.000	0.004	0.786

Table S2. Protein crystallography data collection and refinement statistics (molecular replacement). Values in parentheses are for highest-resolution shell. Each data set was collected from a single crystal.

	SARS-CoV-2 M^{pro} A7T	SARS-CoV-2 M^{pro} E47N	SARS-CoV-2 M^{pro} E47K	SARS-CoV-2 M^{pro} L50F
PDB Entry	8DJJ	8EJ9	8EJ7	8DKZ
**Data collection**				
Space group	C 1 2 1	C 1 2 1	P 1 2_1 1	C 1 2 1
Cell dimensions				
a, b, c (Å)	114.11 53.93 44.71	114.62 54.5 44.85	44.87 54.1 114.01	121.99 82.25 63.88
α, β, γ (°)	90 102.073 90	90 101.186 90	90 101.028 90	90 90.673 90
Resolution (Å)	48.56 – 2.51 (2.6 – 2.51)	28.11 – 2.5 (2.589 – 2.5)	31.39 – 2.3 (2.382 – 2.3)	46.47 – 3.0 (3.107 – 3.0)
Observations	55345 (2927)	62638 (6539)	156339 (16305)	84480 (8708)
R_{merge}	0.09358 (0.4553)	0.135 (0.945)	0.07774 (0.4827)	0.1164 (1.088)
$I / \sigma I$	14.96 (2.99)	8.73 (1.75)	17.05 (4.18)	13.15 (1.68)
Completeness (%)	97.14 (80.04)	97.00 (99.46)	97.08 (98.35)	94.59 (97.41)
Redundancy	6.2 (4.0)	6.7 (7.0)	6.6 (6.8)	6.7 (7.0)
CC1/2	0.997 (0.887)	0.996 (0.785)	0.999 (0.95)	0.998 (0.598)
**Refinement**				
Resolution (Å)	48.56 – 2.51	28.11 – 2.5	31.39 – 2.3	46.47 – 3.0
No. reflections	8968	9246	23448	12109
R_{work}/R_{free}	21.80/27.99	25.52/32.98	20.15/27.04	23.45/31.63
Number of non-hydrogen atoms	2407	2391	4960	4747
Protein	2372	2366	4756	4746
Water	35	25	204	1
B-factors				
Protein	58.32	54.20	37.22	97.69
Water	48.13	50.15	36.75	52.84
R.m.s. deviations				
Bond lengths (Å)	0.002	0.005	0.009	0.011
Bond angles (°)	0.53	0.69	1.05	1.42

Table S3 continued. Protein crystallography data collection and refinement statistics (molecular replacement). Values in parentheses are for highest-resolution shell. Each data set was collected from a single crystal.

	SARS-CoV-2 M^{pro} L89F	SARS-CoV-2 M^{pro} K90R	SARS-CoV-2 M^{pro} P132H	SARS-CoV-2 M^{pro} T190I
PDB Entry	8DKL	8DKJ	8DI3	8DK8
**Data collection**				
Space group	P 1 2_1 1	C 1 2 1	C 1 2 1	P 1
Cell dimensions				
a, b, c (Å)	44.96 53.8 114.76	115.56 54.06 45.13	113.49 52.92 44.85	44.62 54.47 62.81
α, β, γ (°)	90 101.355 90	90 101.079 90	90 102.897 90	115.719 99.579 90.061
Resolution (Å)	31.82 – 1.9 (1.968 – 1.9)	27.5 – 2.11 (2.185 – 2.11)	31.09 – 1.5 (1.554 – 1.5)	43.85 – 2.6 (2.693 – 2.6)
Observations	228777 (21074)	107660 (10613)	219276 (20962)	52297 (4696)
R_{merge}	0.1039 (1.189)	0.1519 (1.65)	0.09456 (1.271)	0.09051 (0.5521)
I / σI	9.02 (1.38)	9.05 (1.16)	11.03 (1.61)	9.36 (2.03)
Completeness (%)	98.00 (95.68)	98.83 (99.30)	98.01 (96.60)	93.92 (92.38)
Redundancy	3.2 (5.2)	6.9 (6.8)	5.4 (5.2)	3.4 (3.3)
CC1/2	0.998 (0.577)	0.997 (0.408)	0.997 (0.6)	0.995 (0.752)
**Refinement**				
Resolution (Å)	31.82 – 1.9	27.5 – 2.11	31.09 – 1.5	43.85 – 2.6
No. reflections	41830	15693	40784	15109
R_{work}/R_{free}	19.98/24.15	19.44/24.69	18.41/21.80	19.73/28.34
Number of non-hydrogen atoms	5062	2423	2691	4827
Protein	4762	2369	2373	4758
Water	300	54	318	69
B-factors				
Protein	36.55	49.20	27.14	56.81
Water	41.17	48.34	37.78	47.59
R.m.s. deviations				
Bond lengths (Å)	0.009	0.008	0.006	0.009
Bond angles (°)	1.09	0.92	0.95	1.22

Table S3 continued. Protein crystallography data collection and refinement statistics (molecular replacement). Values in parentheses are for highest-resolution shell. Each data set was collected from a single crystal.

	SARS-CoV-2 M^{pro} A260V	SARS-CoV-2 M^{pro} G283S	SARS-CoV-2 M^{pro} S284G
PDB Entry	8DKH	8DKK	8DMN
**Data collection**			
Space group	C 1 2 1	C 1 2 1	C 1 2 1
Cell dimensions			
a, b, c (Å)	115.1 53.79 44.78	114.74 54.49 44.88	114.29 54.05 44.78
α, β, γ (°)	90 101.58 90	90 101.029 90	90 101.018 90
Resolution (Å)	28.19 – 1.95 (2.02 – 1.95)	38.45 – 2.0 (2.071 – 2.0)	30.75 – 2.3 (2.382 – 2.3)
Observations	128214 (13358)	126086 (12750)	174492 (17087)
R_{merge}	0.0656 (0.4928)	0.1421 (1.231)	0.1555 (1.272)
I / σI	16.56 (4.05)	10.95 (1.55)	17.40 (5.64)
Completeness (%)	97.20 (97.93)	98.82 (99.02)	97.44 (91.65)
Redundancy	6.6 (7.0)	6.9 (7.0)	14.7 (15.6)
CC1/2	0.998 (0.945)	0.997 (0.771)	0.997 (0.955)
**Refinement**			
Resolution (Å)	28.19 – 1.95	38.45 – 2.0	30.75 – 2.3
No. reflections	19144	18319	11760
R_{work}/R_{free}	24.67/28.79	20.38/24.97	21.11/27.13
Number of non-hydrogen atoms	2537	2504	2457
Protein	2369	2369	2376
Water	168	135	81
B-factors			
Protein	33.01	44.00	37.73
Water	34.91	44.02	37.61
R.m.s. deviations			
Bond lengths (Å)	0.003	0.008	0.002
Bond angles (°)	0.61	0.95	0.53
