# Supplementary material for: SARS-CoV-2 Mpro Protease Variants of Concern Display Altered Viral Substrate and Cell Host Target Galectin-8 Processing but Retain Sensitivity toward Antivirals
Source: ACS Cent Sci. 2023 Mar 21;9(4):696–708. doi: 10.1021/acscentsci.3c00054 (PMC10042146; doi:10.1021/acscentsci.3c00054)
Supplement: Supplementary file 2 — oc3c00054_si_002.pdf [file oc3c00054_si_002.pdf]

oc-2023-00054j.R1

Name: Peer Review Information for "SARS-CoV-2 M^{pro} protease variants of concern display altered viral substrate and cell host target Gal8 processing but retain sensitivity towards antivirals"

First Round of Reviewer Comments

Reviewer: 1
Comments to the Author
This is a very comprehensive biochemical analysis of the effect (both substrate specificity and functional cellular effects as well as inhibitor sensitivity) of mutations of concern on SARS-CoV-2 Mpro activity. This is a timely story with continued concern over SARS-CoV-2 variants. The study is performed thoroughly and rigorously and should be published as is.

Reviewer: 2
Comments to the Author
This paper presents a very thorough structural characterization of variants of concern (VOC) occurring within the SARS-CoV-2 main protease. The paper provides extensive data demonstrating not just that these VOC are still susceptible to the antiviral activity of current protease inhibitors, but also provides an extensive characterization of impact of cleavage of one key human protein gal-8, a host defense protein, on the function of Gal-8. Importantly these data show that Gal-8 cleavage impacts the immunomodulatory effects of Gal-8. Specifically, cleaved Gal-8 had a diminished stimulatory effects on monocytes than the full length Gal-8. This finding is important as it provides the molecular basis for its role as a host defense protein. This is a truly excellent paper and I have no critique about the science itself nor the experimental aspects. My only comments focus on the details of the writing and presentation of the paper.

This paper does an excellent job of investigating the impact of mutation on substrate specificity and on the sensitivity of the protease to antivirals. IN this regard, the work described in this paper is highly related to the work by Celia Schiffer demonstrating the “Substrate Envelope Hypothesis”. A discussion of those concepts is warranted in this manuscript.

I have a suggestion/critique about the title. The data show that the antivirals are potent toward VOC. I believe it should say “retain sensitivity to antivirals” rather than “retain potency towards antivirals.” The current title is mistaken as antivirals are potent against the protease not vice versa (not the protease is potent against antivirals).

In addition, although the paper extensively focuses on characterization of Gal-8 cleavage, that is not reflected at all in the title. I would recommend a title that includes this aspect of the paper.

Column 2 lines 54-55. Clarity could be improved by stating “limited studies have examined the mutations in other essential viral proteins. …”

The coloring in Fig 1B is difficult to parse. The darkest green is 100%. In Alpha, E47N is reported in the text to only comprise 8.6% of the sequences, but it is dark green – essentially indistinguishable from the 100% in the key.

Fig 1 (C) is not defined clearly in the figure legend. It should read (C-E) SARS-CoV-2 Mpro mutants reveal differences in (C) KM, (D) catalytic efficiency (kcat/KM) and (E) Tm values.

“The” is missing on Page 3 in this sentence: The L50F crystal structure revealed loss of charge around THE S2 binding site…, in page 4 in this sentence : THE crystal structure of E47N mutant revealed the change in…, In Fig 3 legend in: and THE reaction was stopped at…,

Fig 2 (F) would be easier to parse if values were given in nM rather than micromolar.

Page 4, Page 6 line 11 and throughout the subscript is missing in KM.

This sentence on page 6 is unnecessary as the data can be readily gleaned from the figure. “We observed a -7 ℃ shift for C160F, C160Y and P184S, -4 ℃ for N180D, -7 ℃ for F185S and -2 ℃ for V186F”

Page 6 lines 38 and 39, right column “At the C-terminal, an ACC (7-amino-4-carbamoylmethylcoumarin) fluorescent tag was attached…” should use the correct phrase “At the C-terminus…”

Page 9 right column Lue should be Leu

Extra text seems to have been inserted into the bottom of page 10 and the top of page 11.

Author's Response to Peer Review Comments:

Dear Dr. Editor,

We would like to thank you for send sending the manuscript entitled “SARS-CoV-2 Mpro protease variants of concern display altered viral and host target processing but retain potency towards antivirals” for review towards publication in ACS Central Science as a research article. We were very pleased with the comments from the reviewers and have addressed all issues in detail below. In the revision we attached a clean copy and a tracked copy. Some edits in the title and addressed required us to edit the supplemental pages so both clean and tracked are provided in word format. Collectively we thank the reviews for helping us strengthen the manuscript.

Reviewer: 1

Recommendation: Publish in ACS Central Science without change.

Comments:
This is a very comprehensive biochemical analysis of the effect (both substrate specificity and functional cellular effects as well as inhibitor sensitivity) of mutations of concern on SARS-CoV-2 Mpro activity. This is a timely story with continued concern over SARS-CoV-2 variants. The study is performed thoroughly and rigorously and should be published as is.

Additional Questions:
Quality of experimental data, technical rigor: Top 1%

Significance to chemistry researchers in this and related fields: Top 1%

Broad interest to other researchers: Top 1%

Novelty: Top 1%

Is this research study suitable for media coverage or a First Reactions (a News & Views piece in the journal)?: No

We thank reviewer 1 for their supportive review.

Reviewer: 2

Recommendation: Publish in ACS Central Science after minor revisions noted.

Comments:
This paper presents a very thorough structural characterization of variants of concern (VOC) occurring within the SARS-CoV-2 main protease. The paper provides extensive data demonstrating not just that these VOC are still susceptible to the antiviral activity of current protease inhibitors, but also provides an extensive characterization of impact of cleavage of one key human protein gal-8, a host defense protein, on the function of Gal-8. Importantly these data show that Gal-8 cleavage impacts the immunomodulatory effects of Gal-8. Specifically, cleaved Gal-8 had a diminished stimulatory effects on monocytes than the full length Gal-8. This finding is important as it provides the molecular basis for its role as a host defense protein. This is a truly excellent paper and I have no critique about the science itself nor the experimental aspects. My only comments focus on the details of the writing and presentation of the paper.

This paper does an excellent job of investigating the impact of mutation on substrate specificity and on the sensitivity of the protease to antivirals. In this regard, the work described in this paper is highly related to the work by Celia Schiffer demonstrating the “Substrate Envelope Hypothesis”. A discussion of those concepts is warranted in this manuscript.

We thank the reviewer for pointing this out. We had in fact in earlier version of the manuscript cited and referred to the substrate-envelope hypothesis, and this must have been removed to

shorten the paper inadvertently. We appreciate the reminder to include this essential link to previous landmark studies.

I have a suggestion/critique about the title. The data show that the antivirals are potent toward VOC. I believe it should say “retain sensitivity to antivirals” rather than “retain potency towards antivirals.” The current title is mistaken as antivirals are potent against the protease not vice versa (not the protease is potent against antivirals).

In addition, although the paper extensively focuses on characterization of Gal-8 cleavage, that is not reflected at all in the title. I would recommend a title that includes this aspect of the paper.

We thank the reviewer for pointing this out and indeed we agree the title could be improved. The title has been adjusted to read: SARS-CoV-2 Mpro protease variants of concern display altered viral and Gal8 host target processing but retain sensitivity towards antivirals

Column 2 lines 54-55. Clarity could be improved by stating “limited studies have examined the mutations in other essential viral proteins. …”

This edit has been included in the manuscript.

The coloring in Fig 1B is difficult to parse. The darkest green is 100%. In Alpha, E47N is reported in the text to only comprise 8.6% of the sequences, but it is dark green – essentially indistinguishable from the 100% in the key.

This figure was a challenge because some mutations are not highly prevalent. We therefore used a log scale. The figure legend has been adjusted to reflect this. Additionally, we changed the color to match the full figure palate.

Fig 1 (C) is not defined clearly in the figure legend. It should read (C-E) SARS-CoV-2 Mpro mutants reveal differences in (C) KM, (D) catalytic efficiency (kcat/KM) and (E) Tm values.

This edit has been included in the manuscript.

“The” is missing on Page 3 in this sentence: The L50F crystal structure revealed loss of charge around THE S2 binding site…, in page 4 in this sentence: THE crystal structure of E47N mutant revealed the change in…, In Fig 3 legend in: and THE reaction was stopped at…,

This edit has been included in the manuscript.

Fig 2 (F) would be easier to parse if values were given in nM rather than micromolar.

This edit has been included in the manuscript.

Page 4, Page 6 line 11 and throughout the subscript is missing in KM.

This edit has been included in the manuscript.

This sentence on page 6 is unnecessary as the data can be readily gleaned from the figure. "We observed a -7 ℃ shift for C160F, C160Y and P184S, -4 ℃ for N180D, -7 ℃ for F185S and -2 ℃ for V186F"

We feel this sentence should remain to allow readers to compare Tm changes and our interpretation of the results.

Page 6 lines 38 and 39, right column "At the C-terminal, an ACC (7-amino-4-carbamoylmethylcoumarin) fluorescent tag was attached…" should use the correct phrase "At the C-terminus…"

This edit has not been included in the manuscript, since this substrate is unique compared to other papers with SARS-CoV2 activity studies. We want the readers to be fully aware that the EDANS-Dabcyl pair was not used in our paper, which will be important especially for comparison with other results.

Page 9 right column Lue should be Leu

This edit has been included in the manuscript.

Extra text seems to have been inserted into the bottom of page 10 and the top of page 11.

This edit has been fixed in the manuscript.

Additional Questions:
Quality of experimental data, technical rigor: Top 5%

Significance to chemistry researchers in this and related fields: Top 5%

Broad interest to other researchers: Top 5%

Novelty: Top 5%

We thank you for consideration of our manuscript for publication in ACS Central Science.
Sincerely,

M. Joanne Lemieux
